# Modelling Charcot-Marie-Tooth disease in a dish reveals common cell type-specific alterations

**DOI:** 10.1093/brain/awab278

**Published:** 2021-08-04

**Authors:** Juliane S Müller, Rita Horvath

**Affiliations:** Department of Clinical Neurosciences, John Van Geest Centre for Brain Repair, School of Clinical Medicine, University of Cambridge, Cambridge, UK

## Abstract

This scientific commentary refers to ‘Induced pluripotent stem cell-derived motor neurons of CMT type 2 patients reveal progressive mitochondrial dysfunction’, by Van Lent *et al.* (doi:10.1093/brain/awab226).

**This scientific commentary refers to ‘Induced pluripotent stem cell-derived motor neurons of CMT type 2 patients reveal progressive mitochondrial dysfunction’, by Van Lent *et al.* (doi:10.1093/brain/awab226).**

Charcot-Marie-Tooth disease (CMT) is one of the most common inherited neurological disorders (∼1 in 2500), and can be caused by mutations in more than 100 nuclear genes.^1^ While CMT leads to abnormalities in both sensory and motor nerves, in hereditary motor neuropathies (dHMN) and hereditary sensory and autonomic neuropathies (HSAN), it is motor neurons and sensory neurons that are affected, respectively. However, there is an overlap across these conditions.

Mitochondria are essential for the health and viability of both motor and sensory neurons and their axons. Processes that disrupt the distribution and transport of mitochondria along axons will likely give rise to peripheral neuropathies. Axonal transport of mitochondria has been extensively studied in a range of mouse models of CMT, along with changes to neurite networks and neuronal excitability. In fact, mouse models have been generated for most CMT-causing genes and have helped reveal a number of the pathomechanisms underlying the disorder.^2^

More recently, the generation of patient-derived iPSC (induced pluripotent stem cell) lines and their differentiation into less accessible cell types such as neurons has become an established approach for studying disease mechanisms. Since 2015, several studies have been published where CMT iPSC lines have been derived from patient fibroblasts or peripheral blood mononuclear cells.^3-6^ These iPSCs were differentiated mainly into motor neurons or neural crest cells, and each publication focused only on one gene. In this issue of *Brain*, Van Lent and co-workers^7^ present the first such study to look at multiple CMT-causing genes and to differentiate the iPSCs into more than one cell type. This approach can help reveal which aspects of the pathomechanism are gene or mutation-specific and which are common to all types of CMT. This information will ultimately be essential for developing therapies for CMT, which can cover multiple CMT subtypes.

Van Lent *et al.*^7^ generated iPSC lines from patients with five different CMT2 mutations—MFN2^R94Q^, NEFL^P8R^, HSPB8^K14N^, HSPB1^G84R^ and HSPB1^P182L^—including those that cause the most common axonal forms of the disorder, as well as from two healthy controls. An isogenic control line was also generated by correcting the *MFN2*^R94Q^ mutation via CRISPR/Cas9 mutagenesis, enabling natural variability to be filtered out. All iPSC lines were differentiated into iPSC-derived motor neurons as well as sensory neurons.

After first confirming previously published gene and mutation-specific features in patient-derived motor neurons (such as neurofilament light inclusions in the *NEFL*-mutated motor neurons), Van Lent and colleagues^7^ focused their attention on identifying pathogenic features common to several CMT2 forms. All CMT2 mutations decreased the length of neurites and the neurite density in motor neurons. Furthermore, all mutations changed the spontaneous electrical activity of motor neurons, albeit not all mutations resulted in the same type of change. While motor neurons carrying *MFN2* and *NEFL* mutations showed an increase in burst rate, the other mutations led to a decrease in activity.

Past studies have demonstrated impaired mitochondrial transport along axons in some forms of CMT, but it is unclear whether this is common to all CMT subtypes. By measuring mitochondrial and lysosomal trafficking in control and patient-derived motor neurons, Van Lent *et al.*^7^ showed that the speed of movement of mitochondria and lysosomes was reduced in all patient cells. In addition, changes in mitochondrial shape (reduced elongation) were seen in all patient-derived motor neurons, and were most pronounced in the *MFN2* and *NEFL* mutant cells.

Van Lent *et al.*^7^ also differentiated the same patient iPSC lines into peripheral sensory neurons, and found that neurite length and mitochondrial and lysosomal transport were affected only in the *MFN2* and *NEFL* mutant sensory neurons. This is consistent with the clinical finding that *HSPB* gene mutations predominantly affect motor neurons in patients, whereas *MFN2* and *NEFL* variants cause both motor and sensory symptoms. This reproduction of cell type-specific findings in the iPSC-derived motor and sensory neurons helps validate iPSC-derived neurons as a disease model.

In a transcriptomic analysis, the authors identified pathways related to the mitochondrial respiratory chain as being downregulated in mutant motor neurons. In accordance with these findings at the RNA level, all patient-derived motor neuron lines also showed reduced oxygen consumption when measured using a Seahorse Analyzer. Reduced oxygen consumption is a reflection of reduced oxidative phosphorylation activity of the mitochondria, although only the *NEFL* mutant cells had a very pronounced reduction of basal and maximal respiration.

The data presented by Van Lent and colleagues^7^ thus reveal abnormalities common to multiple forms of axonal CMT in mitochondrial transport, morphology, gene expression patterns and oxygen consumption, suggesting that mitochondrial dysfunction may be a common hallmark of axonal CMT. Although this work nicely demonstrates the importance of mitochondria in CMT pathogenesis, some important questions remain unanswered: Does the mitochondrial loss-of-function precede the reduction in mitochondrial mobility, or do they occur at the same time? Are both features connected or do they occur independently of each other? Do all mitochondria in the cell show reduced oxidative phosphorylation? Is there a difference between mitochondria in the cell body and those in the axons?

Ultrastructural studies and mitochondrial staining in cells may answer some of these questions. In their discussion, Van Lent *et al.*^7^ hint at the possibility of selective stalling of defective individual mitochondria in axons; an idea that would be interesting to pursue in future studies. It would also be worth defining which mitochondria show the greatest changes in shape. A TMRM (tetramethylrhodamine) assay in live neurons—which only stains mitochondria with an intact membrane potential and thus intact oxidative phosphorylation—could reveal whether the dysfunctional mitochondria are restricted to the axon or are found throughout the whole neuron.

Studying mitochondria in neuronal progenitor cells derived from patient iPSCs may also provide new insights into the molecular mechanisms of peripheral neuropathy. In some of the patient motor neurons studied by Van Lent and colleagues, oxygen consumption was already reduced at Days 24–25. It would be interesting to find out at what point during differentiation mitochondrial defects start to appear. In a recent publication on *SURF1* cerebral organoids,^8^ the mitochondrial defects observed in mature neurons were already evident at the neuronal progenitor stage. As neuronal progenitor cells are small and do not have long neurites, mitochondrial mobility may not be important to their function. However, it would be worth examining whether neuronal progenitor cells of CMT patients also show abnormalities at this earlier stage.

Neurons are post-mitotic cells and lack the ability to divide. The relatively small cell body of the neuron has to maintain the long axon with the aid of axonal transport mechanisms, with mitochondria, lysosomes/endosomes, proteins and RNA (granules) moved from the cell body to the axon terminal (anterograde transport) and from the axonal terminals to the cell body (retrograde transport). This may explain why axonal transport defects seem to be a common disease pathway in CMT, and why targeting axonal transport may be beneficial for a broad range of genetic forms of CMT.

New treatment approaches aim to improve axonal transport with HDAC6 inhibitors by restoring tubulin acetylation.^9^ Van Lent and colleagues^7^ tested another approach, using an inhibitor of the dual leucine zipper kinase (DLK) to modulate stress-induced transcriptional pathways which were affected in the cellular models of CMT. DLK inhibitor treatment improved neuronal function in *MFN2* and *NEFL* mutant sensory and motor neurons, suggesting that common molecular targets are present in both neuronal types (Fig. 1). This illustrates the value of using iPSC-derived neurons to develop treatments for CMT.

**Figure 1:**
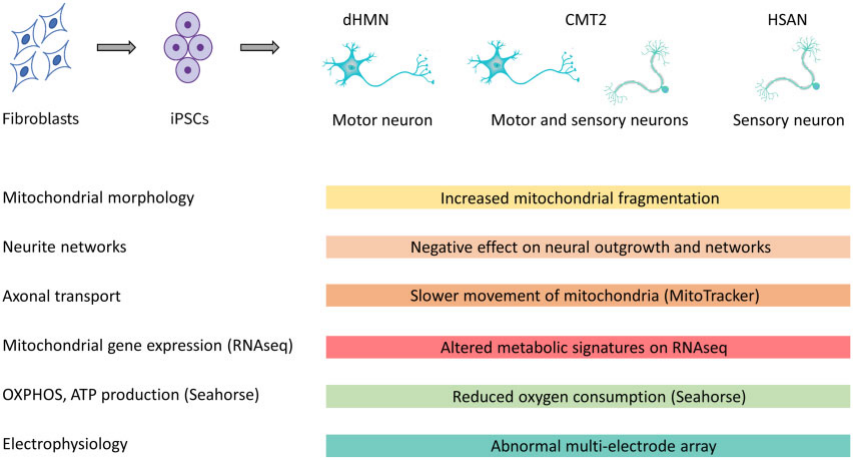
IPSC-derived human neurons in patients with different genetic forms of CMT2, dHMN and HSAN impair the function of motor and/or sensory neurons by altering common pathways, such as mitochondrial fragmentation, neurite outgrowth, movements of mitochondria along axons, cellular metabolism, oxygen consumption and electrophysiological characteristics.
